# Prenatal Diagnosis and Management of Tuberous Sclerosis Complex with Cardiac Rhabdomyoma: A Case Report Highlighting the Role of Sirolimus and Postnatal Complications

**DOI:** 10.3390/diagnostics15141811

**Published:** 2025-07-18

**Authors:** David Asael Rodríguez-Torres, Joel Arenas-Estala, Ramón Gerardo Sánchez-Cortés, Iván Vladimir Dávila-Escamilla, Adriana Nieto-Sanjuanero, Graciela Arelí López-Uriarte

**Affiliations:** 1 Department of Genetics, Facultad de Medicina, Hospital Universitario, Universidad Autónoma de Nuevo León (UANL), Monterrey 64440, Mexico; david.rodriguez@genetica-uanl.mx (D.A.R.-T.); joel.arenas@genetica-uanl.mx (J.A.-E.); 2 Department of Cardiology, Facultad de Medicina, Hospital Universitario, Universidad Autónoma de Nuevo León (UANL), Monterrey 64440, Mexico; rasan_24@hotmail.com; 3 Department of Gynecology and Obstetrics, Facultad de Medicina, Hospital Universitario, Universidad Autónoma de Nuevo León (UANL), Monterrey 64440, Mexico; ivanvladimir@hotmail.com; 4 Department of Neonatology, Facultad de Medicina, Hospital Universitario, Universidad Autónoma de Nuevo León (UANL), Monterrey 64440, Mexico; adrianan75@hotmail.com

**Keywords:** prenatal sirolimus, tuberous sclerosis complex, cardiac rhabdomyomas, necrotizing enterocolitis

## Abstract

**Background and Clinical Significance:** Tuberous sclerosis complex (TSC) is an autosomal dominant disorder caused by pathogenic variants in TSC1 or TSC2. Cardiac rhabdomyoma is a common prenatal finding and can be associated with severe complications, including pericardial effusion. We administered prenatal sirolimus to mitigate pericardial effusion, which led to postnatal complications. **Case Presentation:** A 28-year-old pregnant woman with no significant family history underwent routine fetal ultrasound at 28.1 weeks of gestation, which identified a large right ventricular mass consistent with rhabdomyoma. Further fetal brain MRI revealed cortical-subcortical tubers and subependymal nodules, leading to a clinical diagnosis of TSC. At 30.4 weeks, oral sirolimus (3 mg/day) was started due to the significant pericardial effusion. The effusion remained after treatment, requiring pericardiocentesis at 33.6 weeks. The sirolimus dosage was raised to 6 mg/day at 35.6 weeks, reaching a plasma level of 3.76 ng/mL, but there was no discernible improvement because of the continued fluid accumulation. The mother did not experience any adverse side effects from the procedure. Genetic testing confirmed a pathogenic variant in TSC2 (c.1372C>T). After birth, the neonate received a single dose of sirolimus but subsequently developed necrotizing enterocolitis (NEC), highlighting the potential adverse effects and the need for cautious consideration of treatment options. **Conclusions:** This case illustrates the complexities of managing prenatal tuberous sclerosis complex (TSC). While sirolimus has been explored for fetal cardiac rhabdomyoma and associated complications, its effectiveness in resolving pericardial effusion remains uncertain. Additionally, the development of NEC postnatally raises concerns about the safety of mTOR inhibitors in this context. Further studies are necessary to assess the risks and benefits of this approach in fetal therapy.

## 1. Introduction

Cardiac rhabdomyoma is the most common benign heart tumor in children [1]. It is typically asymptomatic and may occur in isolation or in association with tuberous sclerosis complex (TSC), which can be its first manifestation, even prenatally.

TSC is a genetic disorder with an incidence of 1 in 6000 to 1 in 10,000 individuals. It is caused by heterozygous variants in the TSC1 and TSC2 genes, leading to the abnormal upregulation of the mTOR pathway [1], a signaling system involved in cell growth, proliferation, and metabolism. This dysregulation results in the formation of hamartomatous tumors.

If a patient satisfies one major criterion and two minor criteria, or two primary criteria, TSC is diagnosed primarily based on clinical evidence [2]. Subependymal nodules and cortical tubers are two more possible prenatal markers of TSC.

Of the 66–83% of cardiac rhabdomyomas evident in childhood [1], some can be detected prenatally by fetal echocardiography as single or multiple, rounded, homogeneous, and hyperechoic masses [1,3,4]. A single rhabdomyoma is associated with a 75–80% risk of developing TSC [2,5], while multiple rhabdomyomas increase the risk to 95%. Cardiac rhabdomyomas typically regress spontaneously without the need for treatment [1]. However, some may cause symptoms such as heart murmurs, arrhythmias, heart failure, pericardial effusion, or hydrops. In these cases, early intervention—beginning in the neonatal or even prenatal stage—is crucial [1,2,3].

Only a few cases have been reported on the use of mTOR inhibitors for prenatal treatment in TSC. Everolimus or sirolimus is the standard therapy for rhabdomyomas, even postnatally, which improves symptoms and reduces tumor size [1,3]. At this stage, further research is necessary to gain a deeper understanding of the effectiveness and safety of this approach.

## 2. Case

A 19-year-old healthy woman with no relevant family history presented with a spontaneous pregnancy loss five years ago at 16 weeks of gestation in a twin pregnancy.

**Figure 1:**
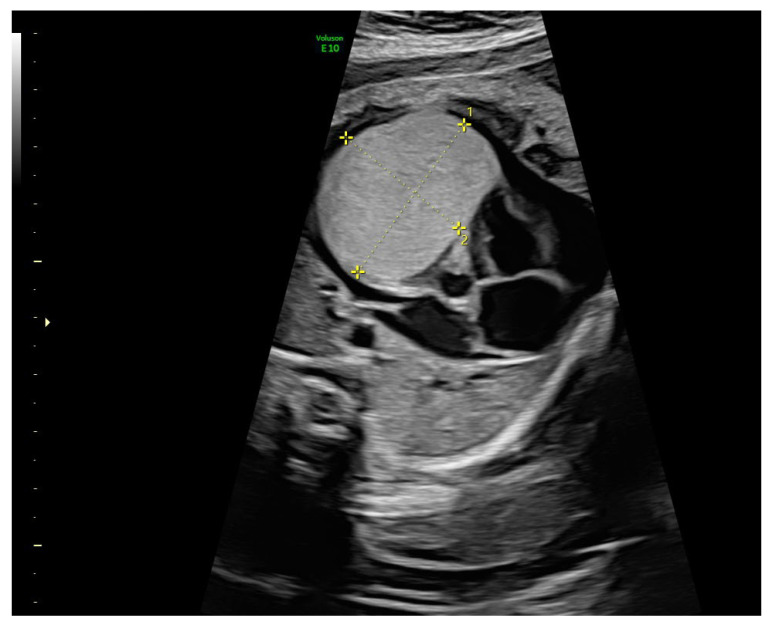
Axial transthoracic view showing a hyperechoic cardiac mass (31.98 × 23.97 × 36.78 mm) with well-defined borders and a mess effect consistent with a cardiac rhabdomyoma. Cardiomegaly and pericardial effusion are also observed.

During her second pregnancy, she was referred to maternal-fetal medicine at 28.1 weeks of gestation after a prenatal ultrasound revealed an intracardiac fetal tumor. Fetal echocardiography identified a hyperechogenic intracardiac mass arising from the anterior portion of the right ventricle, measuring 31.98 × 23.97 × 36.78 mm, without involvement of the ventricular outflow tracts. Severe pericardial effusion was present, raising suspicion of a rhabdomyoma (Figure 1).

At 30.4 weeks of gestation, a fetal MRI showed areas of focal gyral thickening in the bilateral frontal, parietal, and occipital regions of the fetal brain. These areas exhibited mixed signal intensities—some appearing hypointense and others hyperintense on T2, with a mildly hyperintense signal on T1—suggestive of cortico-subcortical tubers. Additionally, the study determined subependymal nodules at the caudothalamic sulcus, the body of the lateral ventricles, and the temporal and occipital recesses of the lateral ventricles. The largest nodule, measuring 8 × 5 mm, was located in the left caudothalamic sulcus adjacent to the foramen of Monro.

In the fetal thorax, the medical team identified a mass arising from the right cardiac wall, which appeared hyperintense on both T1 and T2 sequences. The mass had well-defined margins and measured 34 × 30 × 26 mm. The findings suggested rhabdomyoma, and the mass was associated with pericardial effusion, collectively causing posterior displacement of both lungs.

Observers noted poorly defined peripheral cortical nodules with T2 hypointensity in both kidneys. Given the clinical context, clinicians could not rule out the possibility of angiomyolipomas. Based on these findings, the medical team established a clinical diagnosis of TSC, meeting three primary criteria: multiple cortical tubers, subependymal nodules, and cardiac rhabdomyoma. Both parents underwent clinical evaluation, with no findings suggestive of tuberous sclerosis complex (TSC).

At that time, treatment with sirolimus was initiated at a dose of 3 mg every 24 h, corresponding to an initial dosage of 2 mg/m^2^/day. Follow-up studies at 32.4 and 33.4 weeks of gestation showed no reduction in the size of the cardiac rhabdomyoma. At 33.6 weeks, an increase in fetal pericardial effusion was detected, leading to the decision to perform a pericardiocentesis, which resulted in the drainage of 25 mL of fluid.

At 35.6 weeks of gestation, five weeks after initiating treatment, and with no evidence of a decrease in the size of the intracardiac tumor, the sirolimus dose was increased to 6 mg orally every 24 h. A plasma sirolimus level was 3.76 ng/mL (critical threshold: 25 ng/mL). Despite the absence of tumor reduction, sirolimus was discontinued, even though the mother did not experience any side effects.

**Figure 2:**
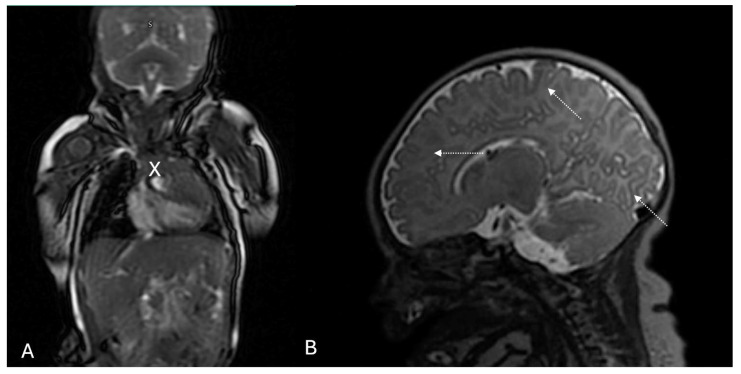
(**A**) Cardiac MRI in FLAIR sequence showing an oval-shaped, solid mass with well-defined borders, homogeneus appearance, and slightly hyperintense signals in T1 and T2, located in the anterior cardiac wall, measuring 33 × 33 × 15 mm (cross mark). (**B**) Additional T2 sagittal and T1 transverce views further confirm the presence of tubers (pointed arrows) and associated structural abnormalities in cortical-subcortical regions.

The male newborn was delivered by cesarean section at 39 weeks of gestation, with a birth weight of 3570 g and a length of 49 cm. A postnatal echocardiogram confirmed the presence of a cardiac rhabdomyoma in the right ventricle, with an MRI measurement of 35 × 33 × 15 mm and no evidence of hemodynamic compromise (Figure 2A). A healthcare provider administered a single oral dose of sirolimus (0.3 mg). On physical examination, they observed no pigmentary skin changes. However, a cerebral MRI revealed subcortical tubers and cell migration abnormalities (Figure 2B)—subsequently, the newborn developed signs of necrotizing enterocolitis, presenting with bloody stools and abdominal distension. Neonatal intensive care initiated antibiotic therapy and fresh frozen plasma. An electrocardiogram showed sinus tachycardia, small R waves in the precordial leads, and deep S waves in the left precordial leads. Due to the gastrointestinal bleeding, they discontinued the mTOR inhibitor.

NGS sequencing of TSC1 and TSC2 identified a nonsense variant in TSC2 (c.1372C>T, p.Arg458*), leading to the production of a truncated protein. At five months of age, an echocardiogram revealed a mass in the right ventricle measuring 3.92 × 4.02 mm, without vascular compromise. The patient underwent an intestinal resection due to the poor progression of necrotizing enterocolitis. Currently, at one year of age, he presents multiple hypopigmented macules on the lower extremities and lumbar region and continues treatment with vigabatrin as a neuroprotector.

## 3. Discussion

Tuberous sclerosis complex (TSC) remains a challenging and multifaceted condition. Advances in treatment options have led to an improved quality of life for individuals affected, along with increased life expectancy and a reduction in morbidity and mortality. TSC characterizes the development of benign tumors in multiple organs, including the brain, kidneys, heart, and skin. Only a few cases have reported prenatal treatment of cardiac rhabdomyoma with mTOR inhibitors, all of which showed a significant reduction in tumor size. However, unlike those reports, the present case showed no such regression; on the contrary, the tumor size increased. The main challenge is predicting tumor behavior with or without treatment, as well as assessing maternal tolerance to the therapy.

Cardiac rhabdomyoma is the most common cardiac tumor identified during the prenatal period, with up to 95% of cases being multiple or associated with a family history of TSC. These tumors are typically diagnosed between 22 and 32 weeks of gestation [6,7]. Potential complications include pericardial effusion, arrhythmias, and outflow tract obstruction. In up to 80% of cases, spontaneous regression can occur without the need for intervention [8,9], or after birth (percentage). It is also important to note that these tumors tend to grow during the second and third trimesters and begin to regress after 32 weeks, which may help guide the optimal timing for therapeutic intervention to maximize efficacy, continuing until the early postnatal period, followed by partial or complete regression during the first year of postnatal life [10,11].

The mammalian target of rapamycin (mTOR) signaling pathway plays a central role in the pathophysiology of tuberous sclerosis complex (TSC), particularly in the regulation of aberrant cell growth and tumorigenesis. Although researchers have made much progress in elucidating the molecular mechanisms underlying this pathway, several aspects remain only partially understood. Central to this regulatory network are the proteins hamartin (encoded by TSC1) and tuberin (encoded by TSC2), which form a functional heterodimer that negatively regulates the small GTPase Rheb, thereby inhibiting mTOR complex 1 (mTORC1) activity. This inhibition prevents the phosphorylation and activation of downstream targets involved in the phosphoinositide 3-kinase (PI3K)/protein kinase B (AKT) signaling cascade, a critical pathway governing cell growth, proliferation, metabolism, and survival [12].

Extracellular stimuli, particularly growth factors, modulate this pathway through receptor tyrosine kinases (RTKs), which activate both the phosphatidylinositol 3-kinase (PI3K)- AKT axis and the extracellular signal-regulated kinase (ERK) pathway, thereby influencing mTOR signaling and cellular homeostasis [12,13]. Disruption in the TSC1/TSC2 complex leads to constitutive activation of mTORC1, resulting in unchecked protein synthesis, cell cycle progression, and ultimately the development of benign tumors, or hamartomas, characteristic of TSC.

Advances in the understanding of these molecular interactions have paved the way for targeted therapeutic strategies to restore balance.

Despite its benefits, several adverse effects, including immunosuppression and an increased risk of infections, have been associated with sirolimus [14,15]. While reports have identified fetal growth restriction as a potential risk, no one has described necrotizing enterocolitis as an adverse outcome.

One of the main challenges in this case was the continuous growth of the cardiac rhabdomyoma despite treatment. Previous reports (Table 1) have described significant tumor reduction following sirolimus therapy, with some instances achieving complete resolution, such as the one reported by Park (2019) [16] during the fetal period. Although there are no established guidelines regarding the optimal timing for initiating mTOR inhibitors, treatment in this case was started at gestational weeks similar to those previously reported, using comparable initial dosages.

Although it may be challenging to determine the exact timing for initiating treatment, the main criterion is the identification of a cardiologic finding, such as pericardial effusion, heart failure, or conduction abnormalities. These clinical signs should guide the decision to start pharmacological therapy. Regardless of the level or type of hospital where the initial evaluation takes place, it is essential to ensure timely referral to specialized centers, where appropriate follow-up can be established and potential complications during treatment can be prevented. In the current landscape, with limited case reports and lack of controlled studies, prenatal sirolimus therapy should be approached as a compassionate use strategy, based on an individualized, multidisciplinary risk–benefit assessment and informed parental decision-making.

A potential factor contributing to the lack of response may have been the failure to achieve adequate maternal drug levels (targeting 10–12 ng/mL) [9,23], as studies have associated levels below 6 ng/mL with treatment failure [17,23]. Additionally, researchers classify sirolimus as a category C drug during pregnancy, and its full spectrum of maternal and fetal adverse effects remains unclear. Nevertheless, doctors observed no adverse effects in the mother during treatment in this case. Decision-makers typically choose between sirolimus and everolimus as their initial therapeutic option. Physicians often prefer sirolimus due to its higher absorption, greater bioavailability, and better safety profile in both prenatal and postnatal settings, while it maintains a similar pharmacodynamic profile to everolimus [6,24,25,26].

Researchers have associated sirolimus with adverse effects, such as immunosuppression and an increased risk of infection [27]. While studies report fetal growth restriction as a potential risk, they have not described necrotizing enterocolitis as a common adverse effect. 

The neonate’s hemodynamic condition was stable until the onset of NEC, with no postnatal echocardiographic evidence of compromise. Although this full-term newborn had an adequate birth weight and did not meet the main risk factors for necrotizing enterocolitis, large rhabdomyomas, such as the one causing pericardial effusion in this case, can lead to poor cardiac output, affecting intestinal blood flow, additionally sirolimus-induced immunosuppression or impaired intestinal perfusion, especially in the context of a large rhabdomyoma, may contribute to the condition. Additionally, inadequate nutrition may have further compromised the immature intestine, exacerbating the condition. [28,29].

While the teratogenic effects of sirolimus remain largely unknown, clinicians must consider other postnatal effects to improve management. In this case, reaching the therapeutic dose failed, and the decision to suspend sirolimus due to the development of necrotizing enterocolitis (NEC) slowed tumor regression more than what is reported in the literature.

Although NEC is a multifactorial condition, sirolimus impacts general immunity, and the combination of the cardiac output defect caused by the rhabdomyoma, along with inadequate nutrition, may have contributed to its development. There are few reports in the literature about the prenatal administration of this medication, and pharmacogenomics may influence its effects on individuals. While the evidence for using mTOR inhibitors is limited, documenting such cases is crucial for identifying potential adverse effects, even among patients receiving appropriate therapy. This documentation will aid in analyzing correlations between tumor size, dosage, and treatment duration. Additionally, clinicians must evaluate potential postnatal adverse effects to understand the patient’s clinical progression fully.

This case underscores the complexity of deciding whether to intervene prenatally. Although the tumor size and pericardial effusion met criteria often associated with poor outcomes, the absence of overt hemodynamic compromise may have justified a conservative approach. 

## 4. Conclusions

The findings from this clinical case underscore the crucial need to document such instances to identify potential adverse effects, even when patients receive appropriate mTOR inhibitor therapy. This documentation is pivotal for analyzing correlations between tumor size, administered dosage, and treatment duration. Moreover, evaluating potential postnatal adverse effects is essential for the patient’s clinical progression.

Documenting these types of decisions, even when outcomes are unfavorable, contributes valuable insight to the evolving discussion of prenatal interventions in TSC.

## Figures and Tables

**Table 1:** Summary of reported cases of prenatal mTOR inhibitor treatment in fetal cardiac rhabdomyomas associated with TSC.

Reference	Drug	Gestational Age at Initiation	Dose	Duration	Route	Fetal/Neonatal Outcomes	Adverse Events
Dagge et al., 2021 [17]	Sirolimus	30 weeks	4 mg/day (adjusted by levels)	3 weeks	Maternal oral	Significant fetal tumor size reduction	Not reported
Ebrahimi-Fakhari et al., 2021 [18]	Sirolimus	28–34 weeks (4 cases)	1.5–4 mg/day (by levels)	2–6 weeks	Maternal oral	Significant tumor reduction in all cases; no fetal arrhythmias	Mild maternal rash in one case; no serious complications
Pluym et al., 2020 [19]	Sirolimus	30 weeks	4 mg/day (adjusted)	4 weeks	Maternal oral	Significant tumor regression; no neonatal complications	Maternal lipid reduction; no significant toxicity
Vachon-Marceau et al., 2019 [20]	Sirolimus	30 weeks	6 mg/day (by levels)	4 weeks	Maternal oral	Tumor reduction; fetal clinical improvement	Maternal lipid reduction; no severe effects
Park et al., 2019 [16]	Sirolimus	30 weeks	2 mg/day	~3 weeks	Maternal oral	Tumor shrinkage; improved signs of obstruction	None reported
Will et al., 2023 [21]	Sirolimus	30 + 4 weeks	3 mg/day (target: 10–15 ng/mL)	3 weeks	Maternal oral	Rapid and complete tumor regression; uncomplicated delivery	Mild maternal nausea; no toxicity
Cavalheiro et al., 2021 [3]	Everolimus	24 weeks	Not specified	4 weeks	Maternal oral	Reduction of subependymal and cardiac lesions	Not reported
Mlczoch et al., 2015 [22]	Everolimus	28 weeks	10 mg/week (divided daily)	~6 weeks	Maternal oral	Marked fetal tumor volume reduction	Mild maternal gastrointestinal discomfort
Present study	Sirolimus	30 + 4 weeks	3 mg/day (adjusted to 6 mg/day) plasma level 6 ng/mL	4 weeks	Maternal oral	Not reduction in rhabdomyoma size; neonatal necrotizing enterocolitis	Not reported

## Data Availability

Data available on request from the authors. The data are not publicly available due to privacy.

## References

[1] Sugalska M., Tomik A., Jóźwiak S., Werner B. (2021). Treatment of Cardiac Rhabdomyomas with mTOR Inhibitors in Children with Tuberous Sclerosis Complex—A Systematic Review. Int. J. Environ. Res. Public Health.

[2] Yıldırım S., Aypar E., Aydın B., Akyüz C., Aykan H.H., Ertuğrul İ., Karagöz T., Alehan D. (2023). Cardiac rhabdomyomas: Clinical progression, efficacy and safety of everolimus treatment. Turk. J. Pediatr..

[3] Cavalheiro S., da Costa M.D.S., Richtmann R. (2021). Everolimus as a possible prenatal treatment of in utero diagnosed subependymal lesions in tuberous sclerosis complex: A case report. Child’s Nerv. Syst..

[4] Montaguti E., Gesuete V., Perolo A., Balducci A., Fiorentini M., Donti A., Pilu G. (2023). A case of massive fetal cardiac rhabdomyoma: Ultrasound features and management. J. Matern.-Fetal Neonatal Med..

[5] Zungsontiporn N., Tantrachoti P., Puwanant S. (2015). Foetal and maternal cardiac rhabdomyomas associated with tuberous sclerosis. Eur. Heart J..

[6] Mustafa H.J., Javinani A., Morning M.L., D’Antonio F., Pagani G., Puranik P.M., Khalil A., Shamshirsaz A.A. (2024). Characteristics and Outcomes of Fetal Cardiac Rhabdomyoma with or Without mTOR Inhibitors, a Systematic Review and Meta-Analysis. Prenat. Diagn..

[7] Hinton R.B., Prakash A., Romp R.L., Krueger D.A., Knilans T.K. (2014). Cardiovascular manifestations of tuberous sclerosis complex and summary of the revised diagnostic criteria and surveillance and management recommendations from the international tuberous sclerosis consensus group. J. Am. Heart Assoc..

[8] Schlaegel F., Takacs Z., Solomayer E.F., Abdul-Kaliq H., Meyberg-Solomayer G. (2013). Prenatal diagnosis of giant cardiac rhabdomyoma with fetal hydrops in tuberous sclerosis. J. Prenat. Med..

[9] Chao A.S., Chao A., Wang T.H., Chang Y.C., Chang Y.L., Hsieh C.C., Lien R., Su W.J. (2008). Outcome of antenatally diagnosed cardiac rhabdomyoma: Case series and a meta-analysis. Ultrasound Obstet. Gynecol..

[10] Racioppi G., Proietti Checchi M., Sforza G., Voci A., Mazzone L., Valeriani M., Moavero R. (2024). Prenatal mTOR Inhibitors in Tuberous Sclerosis Complex: Current Insights and Future Directions. J. Clin. Med..

[11] Lucchesi M., Chiappa E., Giordano F., Mari F., Genitori L., Sardi I. (2018). Sirolimus in infants with multiple cardiac rhabdomyomas associated with tuberous sclerosis complex. Case Rep. Oncol..

[12] Kohrman M.H. (2012). Emerging treatments in the management of tuberous sclerosis complex. Pediatr. Neurol..

[13] Man A., Di Scipio M., Grewal S., Suk Y., Trinari E., Ejaz R., Whitney R. (2024). The Genetics of Tuberous Sclerosis Complex and Related mTORopathies: Current Understanding and Future Directions. Genes.

[14] Hartinger J.M., Ryšánek P., Slanař O., Šíma M. (2022). Pharmacokinetic principles of dose adjustment ofmTORinhibitors in solid organ transplanted patients. J. Clin. Pharm. Ther..

[15] Maria G., Antonia D., Michael A., Kate M., Sian E., Sarah F.E., Mehul D., Pratik S. (2019). Sirolimus: Efficacy and Complications in Children with Hyperinsulinemic Hypoglycemia: A 5-Year Follow-Up Study. J. Endocr. Soc..

[16] Park H., Chang C.S., Choi S.-J., Oh S., Roh C.-R. (2019). Sirolimus therapy for fetal cardiac rhabdomyoma in a pregnant woman with tuberous sclerosis. Obstet. Gynecol. Sci..

[17] Dagge A., Silva L.A., Jorge S., Nogueira E., Rebelo M., Pinto L. (2021). Fetal Tuberous Sclerosis: Sirolimus for the Treatment of Fetal rhabdomyoma. Fetal Pediatr. Pathol..

[18] Ebrahimi-Fakhari D., Stires G., Hahn E., Krueger D., Franz D.N. (2021). Prenatal sirolimus treatment for rhabdomyomas in tuberous sclerosis. Pediatr. Neurol..

[19] Pluym I.D., Sklansky M., Wu J.Y., Afshar Y., Holliman K., Devore G.R., Walden A., Platt L.D., Krakow D. (2020). Fetal cardiac rhabdomyomas treated with maternal sirolimus. Prenat. Diagn..

[20] Vachon-Marceau C., Guerra V., Jaeggi E., Chau V., Ryan G., Van Mieghem T. (2019). In-utero treatment of large symptomatic rhabdomyoma with sirolimus. Ultrasound Obstet. Gynecol..

[21] Will J., Siedentopf N., Schmid O., Gruber T., Henrich W., Hertzberg C., Weschke B. (2023). Successful prenatal treatment of cardiac rhabdomyoma in a fetus with tuberous sclerosis. Pediatr. Rep..

[22] Mlczoch E., Hanslik A., Luckner D., Kitzmüller E., Prayer D., Michel-Behnke I. (2015). Prenatal diagnosis of giant cardiac rhabdomyoma in tuberous sclerosis complex: A new therapeutic option with everolimus. Ultrasound Obstet. Gynecol..

[23] Peron A., Au K.S., Northrup H. (2018). Genetics, genomics, and genotype–phenotype correlations of TSC: Insights for clinical practice. Am. J. Med. Genet. Part C Semin. Med. Genet..

[24] Hurtado-Sierra D., Ramos Garzón J.X., Rojas L.Z., Fernández-Gómez O., Manrique-Rincón F. (2023). Case report: Accelerated regression of giant cardiac rhabdomyomas in neonates with low dose everolimus. Front. Pediatr..

[25] Mao B., Zhang Q., Ma L., Zhao D.-S., Zhao P., Yan P. (2022). Overview of Research into mTOR Inhibitors. Molecules.

[26] Silva-Sánchez M.P., Alvarado-Socarras J.L., Castro-Monsalve J., Meneses K.M., Santiago J., Prada C.E. (2021). Everolimus for severe arrhythmias in tuberous sclerosis complex related cardiac rhabdomyomas. Am. J. Med. Genet. Part A.

[27] Bravo Oro A., Esmer Sánchez M.d.C., Rubio Hernández M.E., Morales Ibarra J.J., Reyes Vaca J.G., Villegas Valdez D.M.M., Gómez Elías C.L. (2020). Respuesta a everolimus en un neonato con Rabdomioma cardiaco asociado con el Complejo Esclerosis Tuberosa. Acta Pediátrica México.

[28] Cai X., Golubkova A., Hunter C.J. (2022). Advances in our understanding of the molecular pathogenesis of necrotizing enterocolitis. BMC Pediatrics.

[29] Kashif H., Abuelgasim E., Hussain N., Luyt J., Harky A. (2021). Necrotizing enterocolitis and congenital heart disease. Ann. Pediatr. Cardiol..

